# P-1486. Comprehensive Approach to *Vibrio Vulnificus*: Integrating Drug Therapy, Diagnostic Modalities, and Targeted Care for At-Risk Populations

**DOI:** 10.1093/ofid/ofae631.1656

**Published:** 2025-01-29

**Authors:** Hashem Haj Ebrahimi, Hiyam Ghneim, Tahsin Farid

**Affiliations:** University of Debrecen, Debrecen, Hajdu-Bihar, Hungary; University of Debrecen, Debrecen, Hajdu-Bihar, Hungary; US Food & Drug Administration, Richmond, Texas

## Abstract

**Background:**

Vibrio vulnificus (VV), is endemic to warm coastal waters where it causes significant foodborne and aquatic illnesses resulting in high mortality and morbidity. There are no FDA-approved treatments or standard management strategies. Here we explore the landscape of VV infections, their characteristics and treatment outcomes.

**Figure 1:**
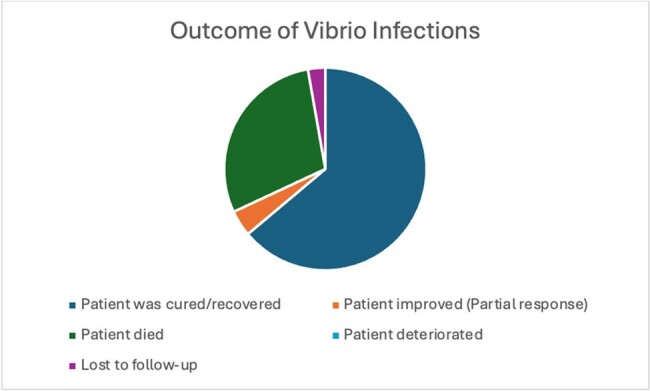
Outcome of Vibrio Vulnificus Infections

**Methods:**

Cases of *VV* from 2014 to 2024 were identified from PubMed and Embase databases, employing pertinent keywords, MeSH terms, and Emtree terms. Articles underwent screening using Rayyan.ai using PRISMA protocol. 72 cases that met inclusion criteria were entered to CURE ID and aggregated data was analyzed.

**Figure 2:**
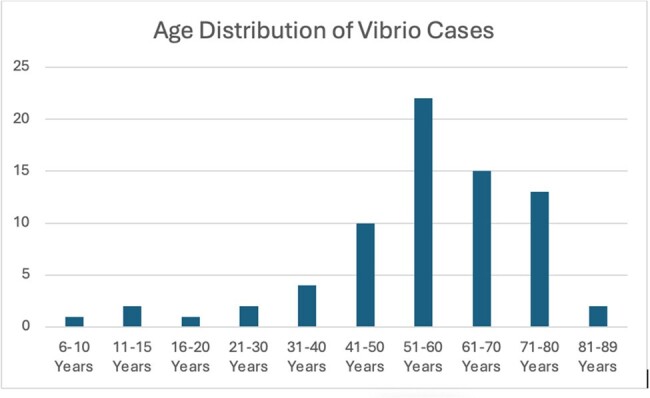
Age Distribution of Vibrio Cases

**Results:**

The outcome distribution of cases are in Figure 1. 46/72 patients recovered while 21 died. Age and gender of patients with VV infections are displayed in Figure 2 and 3 respectively. Most cases occurred in middle aged individuals while there was a male prevalence in cases. Figure 4 displays the countries where most infections occurred. China and the United States had the highest incidence of cases.

**Figure 3:**
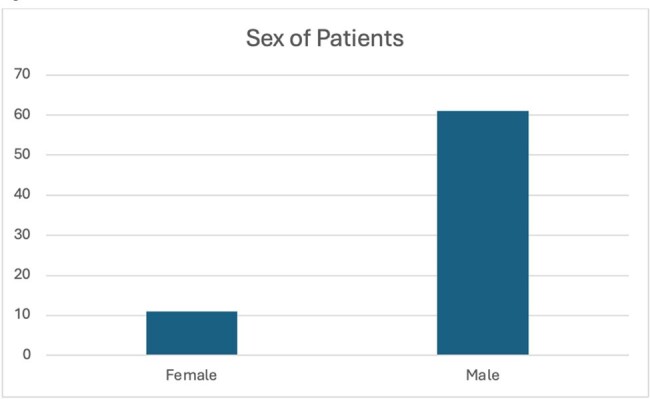
Sex of Patients

**Conclusion:**

The high mortality of VV underscores the importance of finding treatment options for this often-forgotten disease. The age and sex distribution in middle aged males is indicative of the correlation between aquatic activity such as fishing and VV. Males who engage in these activities are at highest risk of contracting VV infections. The geographic distribution of cases underscores countries with warm oceans is where most cases occur. In our data we noticed a prevalence of chronic liver diseases amongst VV patience. We aim to explore this correlation and most effective antimicrobials in a full manuscript.

**Figure 4:**
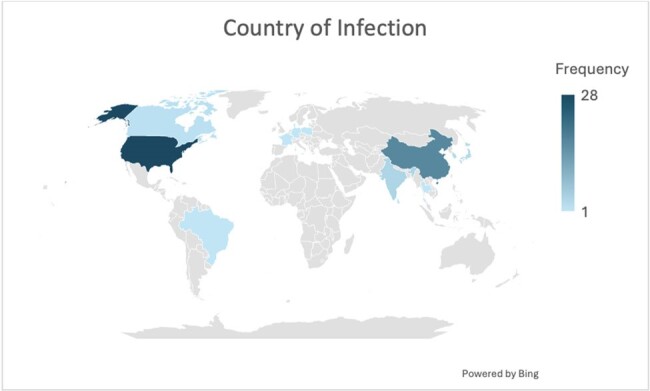
Geographic Distribution of Vibrio Vulnificus

**Disclosures:**

**All Authors**: No reported disclosures

